# At the edge of war: What rapid reinforcement of European defence may mean for military chaplains

**DOI:** 10.1007/s10943-026-02625-2

**Published:** 2026-03-17

**Authors:** Jan Grimell, Alexandra Dierks, Matthias Inniger, Taavi Laanepere, Tatiana Letovaltseva, Tiia Liuski, Laura Mudde, Niklas Peuckmann, Carmen Schuhmann, Janne Aalto, Tabitta Flyger

**Affiliations:** European Research Unit for Military Chaplaincy at Umeå University, Umeå, Sweden

**Keywords:** Military chaplaincy, Military chaplains, Rearmament, War, Moral injury, Europe

## Abstract

Europe’s rapid rearmament in response to Russia’s war against Ukraine marks a significant shift away from the post–Cold War era of demilitarisation and expeditionary operations. Within this evolving security landscape, military chaplains (MCs) in NATO member and partner countries may increasingly find themselves operating on the edge of war. This reflective article asks what this new security reality may mean for military chaplaincy in Europe. Challenging reductive understandings that confine chaplaincy to religion or spiritual practice alone, the article suggests that military chaplaincy can be understood as a profession engaged with existential, moral, and human dimensions of military service. War tends to affect the human person in fundamentally existential ways, and MCs may play a distinctive role in upholding humanity, safeguarding human dignity, and supporting moral orientation in contexts marked by violence, fear, moral conflict, and psychological pressure. The analysis proceeds across four interrelated levels: socio-cultural, organisational, conceptual, and individual. Drawing on interdisciplinary research, the article explores how chaplains may engage in preventive moral work, navigate risks of dehumanisation and radicalisation, and operate within culturally embedded and nationally diverse chaplaincy systems. The article further argues that the capacity to act adequately in wartime may not originate on the battlefield alone, but can be grounded in long-term ecclesial, ethical, educational, and professional formation, as well as sustained interaction between research and practice. Finally, it suggests that effective military chaplaincy may depend on enabling organisational conditions, leadership, and institutional recognition during periods of rearmament and heightened war risk.

## Introduction

The last decades show different tendencies in attention to the practice of military chaplains (hereafter MCs). After the fall of the Berlin Wall in 1989 and the collapse of the Soviet Union in 1991, a long era of disarmament began in most European countries (Moskos et al., 2000). In the subsequent era, many though not all European armed forces gradually decreased in size. At the same time, the shift towards smaller force structures in many countries created conditions conducive to increased voluntarism and professionalisation (Fürst & Kümmel, 2011). Moskos et al. (2000) argued that, in their postmodern model, armed forces became more politically governed, shifted their focus from territorial defence to international operations in distant theatres, and developed a less distinct boundary between the military and civilian spheres. This contributed to a convergence of values that increasingly mirrored those of the surrounding society, including a military role that came to be characterised more and more by that of a soldier–civil servant (Moskos et al., 2000). This era also affected military chaplaincy, not least because demand declined and the reality of war appeared largely absent from everyday military life. At the same time, chaplaincies, to varying degrees, addressed issues of religious diversity and the development of more inclusive multifaith models. Secularism and growing religious plurality emerged as central challenges, prompting new approaches to pastoral and spiritual care and, in turn, reshaping the self-understanding of chaplaincy (Glasner et al., 2023; Liuski & Ubani, 2020; Visser et al., 2025).

### Deployments to remote theatres

International deployments gradually increased for many NATO members and partner countries in Europe, as foreseen by Moskos et al. (2000) in their postmodern model of the armed forces. Not least through the “war on terror” in Afghanistan and elsewhere, this in many respects marked a culmination of joint operations during the postmodern era (Agrell, 2013). This development gave rise to two notable trends.

First, researchers began to observe the emergence of a more radical military culture within deployed units, referred to by some Nordic scholars as a “warrior culture” (Abrahamson, 2011; Edström et al., 2009). Rather than fostering greater integration in terms of civil-military values, this development can be understood as having widened the gap between military and civilian culture and lifeworlds. It also generated a range of challenges related to the transition from military to civilian life, including but not limited to difficulties in identity reconstruction and adaptation to civilian life in a society at peace (Grimell, 2022).

The second trend took shape as emerging research, particularly on moral issues among veterans following operations such as those in Afghanistan, brought renewed attention to the significance of military chaplains. This development is clearly exemplified by Litz et al.’s seminal article on moral injury and moral repair in war veterans (Litz et al., 2009). The concept of *moral injury* has since given renewed relevance to the role of MCs (Carey & Hodgson, 2018; Carey et al., 2016; Graham, 2017; Karle & Peuckmann, 2022; Koenig et al., 2023; Mudde et al., 2025; Nakashima Brock & Lettini, 2012; Wortmann et al., 2017).

The context of international military operations tends to highlight the urgency of MCs and the competence, expertise, and traditions they represent (Stallinga, 2013).

### Military chaplaincy in wartime

In wartime, this urgency becomes even more pronounced, as operational demands, moral stress, and exposure to violence and death further underscore the significance of MCs.

Recent research from wartime Ukraine has clearly illustrated the important role of MCs, and how central their work with morality, ethics, and character formation becomes in a situation that continuously erodes these foundations—indeed, one that erodes what humanity is and what it means to be (Grimell, 2025a, b). MCs act as defenders of humanity in situations where the erosion of values and shifting boundaries of violence are constantly at play. By being present with combat units and elsewhere, they engage directly with these aspects of war and provide care and counselling for existential, spiritual, and religious needs when service members confront the fragility of life and war provoked thoughts and dilemmas (Grimell, 2025c, d).

Not least, MCs also encounter and manage the extensive death on the battlefield as part of their mission. In peacetime military contexts, death can be approached in a more ordered, dignified, and respectful manner. In the referenced research, funerals for military personnel were regarded by many MCs as among the most emotionally demanding parts of their work (Grimell, 2025b).

### The new security reality in Europe

Having long relied on postmodern, small-scale, professionalised, and expeditionary armed forces primarily engaged in distant theatres (Fürst & Kümmel, 2011; Moskos et al., 2000), Europe has, in general, rapidly moved towards an exceptional military rearmament in order to strengthen its defence capabilities, although some countries already possessed a high level of military preparedness. This shift is unfolding at a moment when the democratic rules-based international order and territorial integrity are perceived as acutely threatened, and have therefore returned to the very top of the security agenda. The war in Ukraine and the imminent risk of a wider conflict spreading beyond Ukraine define the situation shaping the ongoing military build-up across Europe and elsewhere.

It is within this transition that MCs serving in NATO member and partner countries in Europe find themselves today. Accordingly, this reflective article asks what this new security reality may mean for MCs.

We aim to develop this reflection on remilitarisation in Europe during a period marked by heightened war risk, and on what this means, or may come to mean, for MCs operating within NATO and partner country contexts. At the same time, while several European countries are currently reforming their chaplaincy services for a variety of reasons, this reflection may offer useful insights into these processes.

This reflection is intended to stimulate thought and discussion across all levels of military chaplaincy, from strategic leadership positions (e.g. Chiefs of Chaplains) to battalion-level practice and other forms of hands-on chaplaincy as a professional craft.

Our analysis proceeds across several interrelated levels:

- first, a broader socio-cultural level;
- second, an organisational level (e.g. national armed forces, churches and faith communities, and NATO);
- third, a conceptual level (e.g. frameworks, concepts, and perspectives);
- fourth, an individual level (e.g. integrity, vulnerability to radicalisation, and competencies).

The reflection is situated in a Northern and Western European context and is written by researchers and MCs affiliated with the newly established European Research Unit for Military Chaplaincy in Sweden. Europe is a vast and diverse context, and our reflection is therefore general in scope, while being primarily situated in the context in which we are professionally engaged.

## Broad socio-cultural level

### Despite similarities, diversity remains

While many of Europe’s armed forces are NATO members and partners operating under a shared defence umbrella, with increasing demands for standardisation and interoperability, responsibility for military chaplaincy services remains national. The field therefore resists broad generalisation and cannot be assumed to be uniform across contexts. On the contrary, we wish to emphasise from the outset that we are acutely aware of substantial cross-national variation, particularly in a European landscape characterised by numerous small- and medium-sized states with distinct cultures and languages. Differences concern organisational arrangements, training and employment structures, as well as purpose, mandate, and assigned tasks. They also reflect the wider societal, cultural, and traditional contexts within which both armed forces and military chaplaincy are situated (Grimell et al., 2025; Pleizier & Schuhmann, 2022).

The cultural context constitutes a fundamentally important point of departure for MCs, since it provides the language, concepts, culturally specific interpretive keys, and meaning-making frameworks through which chaplaincy is practised and understood. Awareness of the significance of culture for chaplaincy as a form of professional craft is particularly evident among some of the world’s most war-experienced chaplains, as seen in wartime Ukraine (Grimell, 2025b, 2025d). The culture in which both the unit and the chaplain are embedded is therefore pivotal to how chaplaincy is shaped, enacted, and received.

This implies, among other things, that anyone seeking to understand what MCs do must recognise that MCs are situated within multiple cultural domains: a broader societal culture; an ecclesial, religious, spiritual, and existential landscape; and a military culture governed by its own distinct logic and interpretive frameworks (Grimell & Letovaltseva, in press). During phases of remilitarisation and in wartime, the software of culture, such as values and normative assumptions, may shift markedly, requiring MCs to navigate these changes with sensitivity and discernment.

## Organisational level

### Expanding armed forces: increased demand

Many European armed forces are currently expanding as part of ongoing rearmament, with new units and formations being established. While some states retain predominantly professional personnel systems, others are reactivating conscription or increasing force volume through forms of compulsory national service. This development implies, among other things, that additional MCs may need to be recruited to support newly established units and formations, at the same time as increasing numbers of recruits, more or less voluntarily, enter military service. It is therefore reasonable to assume that both national and overall populations of MCs will grow in parallel with rearmament in countries where disarmament has previously taken place. Within organisations undergoing expansion, chaplains may encounter cohorts of recruits whose motivations for service, and the spiritual and existential questions evoked by contemporary circumstances and the wider security environment, differ considerably from those shaped during the postmodern era of small expeditionary forces staffed by professional soldiers primarily focussed on international deployments (Fürst & Kümmel, 2011; Moskos et al., 2000).

Today’s young recruits, Generation Z or Gen Z, enter military organisations shaped by a markedly different Zeitgeist than that of the Cold War and the postmodern demilitarisation era during which they were born. Generation Z in Northern and Western Europe is often characterised by digitally shaped identity formation, heightened vulnerability in relation to mental health, and increased uncertainty about the future, while also combining strong individualism with broadly liberal and universalist value orientations (Abu Shehab et al., 2025; Dokoupilová et al., 2024). At the same time, some young people in Europe support illiberal and far-right political currents (van der Brug et al., 2026), underscoring the complexity and heterogeneity of the growing population of young military personnel.

#### The scale of death in full-scale war: challenges at every level

It should also be emphasised that war as such may accelerate the demand for MCs. Research from Ukraine illustrates that MCs may be injured or killed in combat, which can leave units without chaplaincy support for shorter or longer periods (Grimell, 2025b, 2025d). Since recruiting and training MCs is often time-consuming, and because service in combat units and other operational settings requires specific competence and preparedness, this vulnerability should be taken into account in force planning and organisational development.

In wartime, this may require a flexible approach to internal interoperability among MCs within an armed force, including readiness to redistribute or reassign chaplains between branches and units as needed. A certain degree of redundancy in chaplaincy capacity may likewise be understood as a practical means of ensuring operational endurance and addressing gaps arising from compassion fatigue (Stewart, 2012), injury or death among MCs.

From this perspective, it is also important to consider how MCs can recover in a reasonable and sustainable manner during wartime. Research from Ukraine suggests that this is difficult to achieve (Grimell, 2025b). At the same time, it should be acknowledged that a professionalised military chaplaincy service has been established and continues to develop under conditions of full-scale war, which is a remarkable accomplishment in its own right. An MC is expected to care for, counsel, and support a very large number of service members and commanders, often at the battalion level. Yet who supports the MC, and what organisational conditions are needed to enable recovery over prolonged periods marked by suffering and death on the battlefield? Research from wartime contexts suggests that a formal mentoring system may be beneficial in this regard. It also highlights the importance of having a confessor, of caring for and renewing one’s spirituality, of maintaining sound routines (sleep, nutrition, and a healthy lifestyle), and of staying physically fit (Grimell, 2025b).

Amid the intensive remilitarisation currently underway, it is easy to focus primarily on technical matters, material, the establishment of new units, and the training of additional military personnel. It is also crucial to prepare for and train for what may be the most challenging dimension of war: death. The estimated death toll in Ukraine is staggering, and if anything must be rehearsed thoroughly in peacetime, it is how military burial services are to operate in wartime in a dignified, reverent, and respectful manner, in line with the First Geneva Convention (1949), notably Articles 15 and 17, which require parties to search for and collect the dead, prevent their desecration, and ensure honourable burial and the respectful maintenance of graves. The realities of the battlefield in the war in Ukraine challenge the traditional performance of these tasks (Grimell, 2025b). This poses a significant challenge for military chaplaincy services, which must consider how to adapt to a battlefield environment in which the recovery of wounded or fallen soldiers is extremely dangerous and, in many cases, impossible. These developments raise important questions about the adaptation of chaplaincy roles, practices, and support mechanisms in modern high-intensity warfare and underscore the need for further empirical research on how chaplaincy services can operate effectively under such conditions. Ensuring that this system functions effectively is crucial for sustaining ethical and moral dimensions when military personnel are killed in action. This is closely connected to fundamental human concerns such as experiences of dignity, respect, meaning, humanity, and grief.

### Churches and faith communities: planning and preparing for increased demand

The provision of MCs to armed forces varies considerably across national contexts. In many countries, though by no means all, a substantial share of MCs is recruited from churches and other faith communities. Employment arrangements likewise differ, ranging from full-time positions within the armed forces to various hybrid models in which chaplains are employed by a church or other faith community and serve part-time as MCs while simultaneously holding parish-based or equivalent religious appointments (Grimell et al., 2025; Liuski & Grimell, 2022).

An increased demand for MCs may, particularly in contexts where churches and faith communities already face shortages of clergy and religious personnel in civilian settings, generate competing claims over the same category of professionals—this can currently be clearly observed in Germany and elsewhere. For churches and faith communities, it is naturally a priority to ensure that their own ministries and services remain sustainable and functional. This must also be understood in relation to wartime conditions, where large-scale civilian needs for clergy and religious representatives are likely to intensify, not least in response to death, grief, and suffering within society at large (Grimell, 2025b, 2025d). In the event of war, this may therefore result in reluctance to release clergy and religious personnel to military chaplaincy services.

At the same time, parish clergy and other religious representatives may find it difficult to leave their congregations and communities at a time when these are acutely dependent on their leadership, support, and presence. This tension is further intensified by the fact that armed forces in a wartime scenario are likely to require additional MCs, as existing chaplains, to varying degrees, may be injured or killed (Grimell, 2025b).

Wartime thus produces a delicate balance between resources and demand, which will of course differ across national contexts. For this reason, such personnel supply challenges need to be acknowledged already in peacetime, when there is still scope to plan, size, and build preparedness for these contingencies.

#### Churches and theology rethinking peace ethics in a time of war

These developments do not only affect churches and religious communities in terms of personnel. The need for rearmament also means that churches and theology are rethinking peace ethics in a new light and under changed circumstances. The development of rearmament is thus changing not only MC, but also the churches themselves. A case in point is the traditionally pacifist Protestant church in Germany which has just published a new “Friedensdenkschrift”, i.e. a memorandum of peace ethics (Evangelischen Kirche, 2025). While upholding the motif and ideal of peace in justice, the focus has noticeably shifted, especially in comparison with the previous Friedensdenkschrift of 2007. One of the first necessities acknowledged now is the duty of the state to protect its citizens from violence. The role of the armed forces is defined as fulfilling this duty to protect. While this role is defined as strictly defensive, the ethical assessment of what the armed forces do appears in a different light. In consequence, ministers of the church serving as MC in the armed forces are viewed with less ambivalence than before.

### At the NATO research level: increased interest in military chaplaincy

Over the past four years, following the outbreak of the war in Ukraine, interest within the NATO Science and Technology Organization (STO), particularly in the Human Factors and Medicine (HFM) panel, has grown regarding the role of military chaplaincy and the spiritual dimension. This development has become visible through several research initiatives within STO HFM.

One illustrative example is Research Task Group (RTG) 352 on *Moral Challenges in the Future Security Environment* (running between 2022 and 2026), currently in its final phase prior to publication of the concluding report. Within this RTG, the role of MCs and their contribution to the moral continuum (i.e. ranging from conscience and integrity to moral stress, moral distress, and moral injury) has been examined and articulated.

Further evidence of this emerging research agenda can be found in Exploratory Team (ET) 209 on *The Spiritual Dimension in Military Health* (conducted between 2023 and 2024), which in turn led to Workshop 394 on *Spiritual Dimension in Military Health and Resilience*, held in Stockholm in 2025. A similar orientation is also evident in the newly established Research Task Group 408 on *Chaplains and Spiritual-Existential Wellbeing* (planned to run between 2025 and 2028), which places particular emphasis on MCs’ role in relation to spiritual and existential dimensions of wellbeing.

Taken together, these initiatives illustrate a growing and increasingly institutionalised interest in military chaplaincy within NATO’s research organisation. Notably, this interest is being developed within a multinational research environment composed of appointed researchers, subject matter experts, and practitioners from NATO member states and partner nations.

For MCs, the growing interest within NATO STO HFM in military chaplaincy and the spiritual–existential dimension carries several clear implications, as we see it.

#### Recognition

First, it might signal that the chaplain’s role is increasingly recognised as part of military capability, especially in relation to morale, ethical conduct, and the need to address spiritual and existential dimensions in military settings and on the battlefield, across the full deployment cycle, including pre-, during-, and post-deployment contexts.

#### Professionalisation

Second, it points towards heightened expectations of professionalisation, where research engagement, methodological development, and more evidence-informed practice are likely to become increasingly important.

#### Harmonised terminology

Third, it suggests a move towards greater interoperability across national contexts, not through standardisation of chaplaincy as such, but through the potential emergence of shared concepts, models, and arenas for collaboration. A harmonised terminology that can function as a mission effective glossary to align language among chaplains, mental health professionals, commanders, and researchers, to the extent possible.

#### Deeper understanding

Fourth, these research initiatives enable a broader and deeper understanding of what MCs may do and contribute. In particular, chaplaincy approaches may have a wider scope than support for religious believers alone and can therefore be understood as a resource for the unit as a whole, especially in relation to existential and moral challenges (Schuhmann et al., 2023). At the same time, it must be acknowledged that the frameworks, purposes, and tasks that shape chaplaincy approaches differ considerably between countries, ranging from broadly existential orientations that encompass all military personnel to more faith- and religion-specific forms of support, which in practice may entail confessional or humanist orientations and more clearly faith-niched chaplaincy roles.

#### Risk of instrumentalisation

Fifth, a growing understanding of military chaplaincy and a potentially expanded mandate may also carry the risk of instrumentalisation, whereby chaplaincy becomes valued primarily for its organisational utility rather than for its independent and occasionally critical role. It is therefore important to safeguard chaplaincy integrity throughout this ongoing development. (Grimell, 2024; Peuckmann, 2022). This relates to the social dynamics of powerful institutions such as the military. While these effects cannot be avoided completely, it is important to reflect on them to prevent an uncritical conflation of military and chaplaincy roles. Moreover, much of military chaplaincy work cannot readily be captured through shared concepts or standardised frameworks. Instead, it is deeply embedded in culture and tradition, and shaped by the spiritual and existential communities to which chaplains belong, as well as by chaplaincy understood as a form of professional craft and practical, embodied skill (Grimell et al., 2025).

## Conceptual level

### Nationally embedded frameworks and concepts of military chaplaincy

MCs are typically governed by distinct frameworks, regulations, purposes, and mandates across national contexts (Grimell et al., 2025). These arrangements are shaped by factors ranging from national legislation to internal regulations and policies within armed forces, church ordinances, as well as norms and rules within different ecclesial and religious traditions. Chaplaincy is further influenced by the societies, cultures, and traditions within which military chaplaincy services are embedded and from which they emerge. Although the everyday “craft” of chaplaincy on the ground may, at first glance, appear broadly similar across national borders, this similarity should not obscure the substantial diversity of European chaplaincy systems. One implication is that concepts such as interoperability must be applied with caution when discussing military chaplaincy in a European context (Grimell and Letovaltseva, In press). There are also limits to what can be meaningfully translated or transferred conceptually, both between different armed forces and within chaplaincy services inside the same country—for instance, where chaplaincy is organised as a faith-specific niche system primarily serving a particular group of service members.

Given differences in frameworks, cultures, traditions, and chaplaincy approaches, generic concepts likewise need to be interpreted and applied with caution. It is crucial to underscore that the strengths of chaplaincy, and what often constitutes the core of excellent chaplaincy practice, are closely connected to the culture, tradition, and practices already established within a given context (Grimell et al., 2025). Military chaplaincy should therefore be understood and developed through a culturally sensitive lens. Indeed, seemingly well-established notions such as “spirituality” (Pargament, 2011; Pargament & Sweeney, 2011) may not translate smoothly when defined in a North American context (e.g. in terms of meaning, purpose, the search for significance, community, self, identity, and transcendence) and subsequently applied in a Nordic socio-cultural setting (e.g. Sweden and Finland). In such contexts, the term “existential” may in practice better capture much of what is often meant by “spirituality” (Grimell et al., 2025).

### Deepening reflection on the existential and theological–ethical challenges of war

At a time when the war in Ukraine is now in its fourth year, while other European states simultaneously intensify both military and civilian defence build-ups, it becomes important to reflect on what these developments mean for theological perspectives with particular relevance to war. Undoubtedly, war—and all that follows in its wake—raises substantial and pressing theological questions for churches and religious traditions, something the Second World War clearly demonstrated in the European context. Yet what contemporary rearmament and war mean theologically remains relatively unclear and underexplored, pointing to the need for further research that addresses theological questions directly relevant to wartime realities. This does not primarily concern classical theological debates about whether war can be justified (Holmes, 2005) or eliminated (Hauerwas, 2001), but rather questions of the kind grappled with by Bonhoeffer (1995a, 1995b, 1997; also see Fowl & Jones, 1998), such as war’s existential and theologico-ethical challenges of resistance and submission, responsibility and guilt, and the will to act rightly in an evil time. Additionally, it is emphasised that these questions are also important to address from non-religious perspectives.

### Moral injury in war: preventive perspectives

Moral injury constitutes another key conceptual contribution (Carey & Hodgson, 2018; Carey et al., 2016; Graham, 2017; Koenig et al., 2023; Litz et al., 2009; Mudde et al., 2025; Wortmann et al., 2017). It offers an analytical and practical framework that was neither established in modernity nor in the postmodern era of demilitarisation, yet has become increasingly influential today. Much of the research on moral injury has emerged in post-military contexts. The concept was coined by Shay and Munroe (1998) in the mid-1990s through their work with US Vietnam veterans. By contrast, there remains relatively limited research on how moral injury functions, and how the concept should be applied, in the midst of ongoing war. As a result, our current understanding of moral injury “in war” remains underdeveloped however progress is being made given the recent recognition of moral problems and subsequently moral injury as a supplementary condition among active military personnel and veterans, which is now noted within the Diagnostic and Statistical Manual for Mental Health Disorders (DSM-5-TR; Mattson et al, 2025).

Nevertheless, moral functioning is critical in war, not least in terms of preserving one’s moral compass and avoiding a descent into moral dehumanisation. This has been a substantial and ongoing focus in the work of Ukrainian military chaplains (Grimell, 2025b). Their efforts may be understood as a form of preventive moral injury work during active warfare, aimed at limiting moral transgressions as far as possible in a context where war continuously presses morality, ethics, and conduct towards the margins (Grimell, 2025d).

### Defenders of humanity: a unifying concept for military chaplaincy in war

This leads to the concept of humanity and humaneness. Ukrainian MCs have described themselves as defenders of the human within military personnel, as defenders of humanity, and as protectors of human values (Grimell, 2025b, d). Perhaps this concept could serve as a unifying cross-cultural mission and shared orientation for MCs across European armed forces: to defend the human within the military person and to uphold human values in an inhumane situation. War has a tendency to erode the social and moral structures that otherwise regulate everyday life in peaceful societies, including the normative expectations of rules-based liberal democratic life and fundamental principles of equality.

Military chaplaincies in Europe are organised in markedly different ways, and the strength of each chaplaincy is closely tied to its specific national and institutional context. Consequently, models cannot simply be transferred or replicated from one country to another. At the same time, military chaplaincies across Europe face a set of shared challenges. There is a growing need for deeper common theological, ethical, and practical reflection on fundamental issues such as evil, violence, resistance, moral injury, and the protection of human dignity under extreme conditions. Across all national contexts, military chaplaincy carries a distinct operational responsibility: to help safeguard humanity, support moral orientation, and strengthen ethical awareness among service members. Closer cooperation between military chaplaincies and researchers across countries can provide valuable guidance for addressing such conceptual questions.

## Individual level

### Integrity amid militarisation and emerging radicalisation

MCs currently find themselves in the midst of an unprecedented rearmament on the brink of war. This rearmament is feverish and intense, driven both by a threat of war from the East—manifest in the war in Ukraine—and by clear signals that Europe must assume greater responsibility for its own security. At the same time, the rules-based democratic order in the Western world is wavering.

In this context—and more specifically within military organisations shaped by military culture—MCs carry out their work. Military culture is characterised, among other things, by hierarchy, discipline, loyalty, cohesion, the logic of combat and killing, ideals of strength and performance, and a pronounced male dominance (Edström et al., 2009; French, 2005; Huntington, 1957; Strachan, 2006). Although the assignments of MCs may differ across armed forces depending on frameworks, purposes, and mandates, they all represent something other than the fighting organisation, while formally remaining non-combatants (even if some may be armed in certain countries).

However, no one operating within a military organization is immune to military culture (Verrips, 2006; Zimbardo, 2008). Everyone in this context is influenced by its values, meaning-making processes, and practices. For the integrity of the mission—and in order to preserve the important perspectives that MCs bring from their traditions—it is therefore crucial to establish strategies and methods that allow them to be shaped by the ongoing rearmament only within reasonable limits (Grimell, 2024).

A potential state of war would be even more likely to produce radicalising and dehumanising effects on all those in or near the battlefield (Verrips, 2006; Zimbardo, 2008). For MCs, this entails a particular need to remain anchored in their principles and values—for example, compassion and shared humanity—in a situation that tends, in itself, to erode and dissolve rules-based orders of social coexistence (Grimell, 2025b–d). Radicalising and dehumanising processes are not confined to the battlefield but can also be observed in contemporary Western societies at social and political levels, a concern addressed, among others, in NATO HFM RTG 278 on *Preventing and Countering Radicalisation to* Violence (conducted between 2017 and 2020) and by Atuel and Castro (2025). From the perspectives of moral resilience, professional military ethics, and human factors research, such dynamics may influence soldiers’ values, decision-making, and interpersonal relations well before deployment. This indicates that military chaplaincy may have a role to play already in peacetime by supporting ethical reflection, moral preparedness, and the cultivation of professional norms that promote cohesion and responsible conduct in high-stress operational environments.

### Integrating well-established and emerging competencies

From a competency perspective, there are several key aspects to consider for MCs in the current situation. The work of sustaining ethics, morality, and character in war appears central, both in safeguarding the human dimension of service members and in upholding humanity on the battlefield. Education and training that strengthen these competencies are likely to benefit the mission, the unit, and the individual, before, during, and after deployment.

Brutality, trauma, suffering, death, and grief are unavoidable realities of war. For this reason, deepening competence and practical craft experience in precisely these areas, ranging from ceremonies, memorial services, and grief support to wisdom and moral guidance, as well as the ability, in the inferno of war, to uphold what is reverent, dignified, and respectful, will be essential in wartime. At the same time, it should be emphasised that education can only prepare individuals to a certain extent. The true school, at least according to Ukrainian MCs, is the one that takes place on the battlefield (Grimell, 2025b).

### Support initiatives

If no formal mentoring system is established within the organization, it may therefore be important to identify a mentor and confessor already in peacetime, recognizing that such relationships may need to be sustained during war. Being a MC during wartime is extremely demanding, bringing into focus both the maintenance of one’s spirituality and the need to sustain healthy routines related to sleep, nutrition, and physical activity (Grimell, 2025a, 2025b). It may also be beneficial to engage in structured exchanges with Ukrainian MCs in order to deepen insight and understanding and to draw on hard-won lessons learned.

In some European countries, such as Belgium, parallel initiatives seek to (re)connect national chaplaincy services across sectors, including prisons, airports, and healthcare, to establish a form of national “intra-operability” in preparation for wartime conditions.

## Concluding remarks

The acceleration of international deployments in the early 2000s, further intensified by the brutal realities of Russia’s war against Ukraine, has re-actualised the significance of MCs. Their role must be understood far beyond reductive views that confine chaplaincy to religion or spiritual practice alone. Chaplaincy work exceeds such simplified interpretations.

In the following overview, we outline our reflections on what the rapid reinforcement of European defence may mean for military chaplains operating at the edge of war (Table 1).

**Table 1:** Overview of analytical levels and key themes

Levels	Key reflective themes
Broad socio-cultural	Despite similarities, diversity remains
Organisational	Armed Forces: Expansion, rising demand, and the scale of death in full-scale war challenging all levels Churches and faith communities: Preparing for increased demand and rethinking peace ethics in wartime NATO research context: Increased interest in military chaplaincy, recognition and professionalisation, harmonised terminology, deeper understanding, and risks of instrumentalisation
Conceptual	Nationally embedded frameworks and concepts of military chaplaincy Deepening reflection on the existential and theological**–**ethical challenges of war Moral injury in war: preventive perspectives Defenders of humanity: a unifying concept for military chaplaincy in war
Individual	Integrity amid militarisation and emerging radicalization Integrating well-established and emerging competencies Support initiatives

The impact of war on the human person is fundamentally existential, and the work of MCs is therefore also about upholding humanity, safeguarding human dignity, and attending to the human dimension of military service—particularly in contexts marked by violence, fear, moral conflict, and intense psychological pressure.

While it is sometimes suggested that war constitutes the ultimate school for chaplains, the capacity to act adequately on the battlefield does not originate there. Rather, it is grounded in deep and sustained forms of formation and practice that encompass the full breadth of human life: rootedness in ecclesial, religious, and spiritual traditions; rigorous education; ethical reflection; mentoring; pastoral formation; professional training; theoretical study in peacetime; interdisciplinary collaboration; and research.

The interaction between research and practice is crucial. Through scientific methods and practice-oriented inquiry, such collaboration contributes to the development of professional competencies and anticipatory capacities, enabling chaplains to engage thoughtfully with emerging moral and human challenges. At the same time, it empirically substantiates the significant added value of military chaplaincy in contexts of conflict and war.

Finally, the ability of MCs to carry out their work effectively is also contingent upon military and civilian structures, leadership, and institutional understanding of chaplaincy’s potential—namely, the conditions that allow chaplains to exercise their role to its fullest extent.

## Data Availability

The data supporting the findings of this study are included within the article.

## References

[CR1] Abrahamson, B. (2011). *Kärnvärden och den militära framgångsformeln: Huset som Clausewitz byggde* [Core values and the military recipe for success: The house that Clausewitz built] (Report from the Swedish Defense University). Försvarshögskolan, Stockholm, Sweden.

[CR2] Abu Shehab, A. H. I., Ciubară, A. B., Burlea, S. L., Doina Carina, V., Grigoraș, M., Buicu, C. F., & Ciubară, A. (2025). From Instagram to TikTok: How social media fuels eating disorders, anxiety, depression, and insomnia in Gen Z. *European Psychiatry,**68*(Suppl 1), S380–S381. 10.1192/j.eurpsy.2025.797

[CR3] Agrell, W. (2013). *Ett krig här och nu: Sveriges väg till väpnad konflikt i Afghanistan [A war here and now: Sweden’s path to armed conflict in Afghanistan]*. Bokförlaget Atlantis AB.

[CR4] Atuel, H. R., & Castro, C. A. (2025). The 3T model of military veteran radicalization and extremism: exploring risk factors and protective strategies. *Frontiers in Sociology,**10*, 1500774. 10.3389/fsoc.2025.1500774 40144280 10.3389/fsoc.2025.1500774 PMC11937086

[CR5] Bonhoeffer, D. (1995a). *Ethics*. Touchstone.

[CR6] Bonhoeffer, D. (1995b). *The cost of discipleship*. Touchstone.

[CR7] Bonhoeffer, D. (1997). *Letters and papers from prison* .E. Bethge, I. Best, L. E. Dahill, R. Krauss, N. Lukens and Trans (Eds).). New York: Touchstone.

[CR8] Carey, L. B., & Hodgson, T. J. (2018). Chaplaincy, spiritual care and moral injury: considerations regarding screening and treatment. *Frontiers in Psychiatry,**9*, 619. 10.3389/fpsyt.2018.00619 30568605 10.3389/fpsyt.2018.00619 PMC6290645

[CR9] Carey, L. B., Hodgson, T. J., Krikheli, L., Soh, R. Y., Armour, A.-R., Singh, T. K., & Impiombato, C. G. (2016). Moral injury, spiritual care and the role of chaplains: An exploratory scoping review of literature and resources. *Journal of Religion and Health,**55*(4), 1218–1245. 10.1007/s10943-016-0231-x 27094705 10.1007/s10943-016-0231-x

[CR10] Geneva Convention. (1949). Geneva Convention (I) for the Amelioration of the Condition of the Wounded and Sick in Armed Forces in the Field, August 12, 1949, 75 U.N.T.S. 31.

[CR11] Dokoupilová, L., Cogiel, A., & Fero, M. (2024). Values as motivating factors for representatives of generation Z in the Czech Republic and Slovakia within the European context. *Frontiers in Psychology,**15*, 1404354. 10.3389/fpsyg.2024.1404354 39021649 10.3389/fpsyg.2024.1404354 PMC11252541

[CR12] Edström, H., Lunde, N. T., & Haaland Matlary, J. (Eds.). (2009). *Krigerkultur i en fredsnasjon [Warrior culture in a peace nation]*. Abstrakt Forlag.

[CR15] Evangelischen Kirche. (2025). Welt in Unordnung – Gerechter Friede im Blick. Evangelische Friedensethik angesichts neuer. Leipzig, Germany: Evangelische Verlagsanstalt.

[CR13] Fowl, S. E., & Jones, L. G. (1998). *Reading in communion*. Wipf and Stock Publishers.

[CR14] French, S. (2005). *The code of the warrior: Exploring warrior values past and present*. Rowman & Littlefield Publishers.

[CR16] Fürst, H., & Kümmel, G. (Eds.). (2011). *Core values and the expeditionary mindset: Armed forces in metamorphosis*. Nomos.

[CR17] Glasner, T., Schuhmann, C., & Kruizinga, R. (2023). The future of chaplaincy in a secularized society: A mixed-methods survey from the Netherlands. *Journal of Health Care Chaplaincy,**29*(1), 132–144. 10.1080/08854726.2022.2040894 35189782 10.1080/08854726.2022.2040894

[CR18] Graham, L. K. (2017). *Moral injury: Restoring wounded souls*. Abingdon Press.

[CR19] Grimell, J. (2025d). Military chaplaincy in Ukraine: Defenders of humanity. *Australian Army Chaplaincy Journal*, 9–27. https://www.army.gov.au/about-us/army-corps/royal-australian-army-chaplains-department

[CR20] Grimell, J., & Letovaltseva, T. (In press). Holy mission or mission impossible? Military chaplaincy interoperability in NATO: Case Studies of Sweden and Belgium. *Health and Social Care Chaplaincy*.

[CR21] Grimell, J. (2022). *The invisible wounded warriors in a nation at peace: An interview study on the lives of Swedish veterans of foreign conflicts and their experiences with PTSD, moral injuries, and military identities*. Lit Verlag.

[CR22] Grimell, J. (2024). Understanding the presence of military priests conducting Military Soul Care in the Swedish armed forces: A medical sociological perspective. *Frontiers in Sociology,**9*, 1408067. 10.3389/fsoc.2024.1408067 39678313 10.3389/fsoc.2024.1408067 PMC11638914

[CR23] Grimell, J. (2025a). Ukrainian military chaplaincy in war: An introduction. *Health and Social Care Chaplaincy,**12*(2), 106–132. 10.1558/hscc.31916

[CR24] Grimell, J. (2025b). Ukrainian military chaplaincy in war: Lessons from Ukraine. *Health and Social Care Chaplaincy,**12*(2), 133–164. 10.1558/hscc.31917

[CR25] Grimell, J. (2025c). Understanding Ukrainian military chaplains as defenders of the human soul. *Frontiers in Sociology,**10*, 1559023. 10.3389/fsoc.2025.1559023 40144279 10.3389/fsoc.2025.1559023 PMC11936900

[CR26] Grimell, J., Letovaltseva, T., Aalto, J., & De Ceuster, H. (2025). Commanding with compassion: Harnessing the potential of military chaplains within the NATO structure. *Frontiers in Psychiatry,**16*, 1599662. 10.3389/fpsyt.2025.1599662 40521594 10.3389/fpsyt.2025.1599662 PMC12162935

[CR27] Hauerwas, S. (2001). Should war be eliminated? A thought experiment. In J. Berkman & M. Cartwright (Eds.), *The Hauerwas reader* (pp 392–425). Durham, NC: Duke University Press. (Original work published 1984)

[CR28] Holmes, A. F. (Ed.). (2005). *War and Christian ethics: Classic and contemporary readings on the morality of war*. Baker Academic.

[CR29] Huntington, S. (1957). *The soldier and the state: The theory and politics of civil-military relations*. The Belknap Press of Harvard University Press.

[CR30] Karle, I., & Peuckmann, N. (2022). In the shadow of the operation. Moral and spiritual injuries as new challenges for pastoral care in the German Armed Forces. In S. Pickard, M. Welker, & J. Witte (Eds.), *The impact of military on character formation, ethical education, and the communication of values in late modern pluralistic societies* (pp. 153–166). Evangelische Verlagsanstalt.

[CR31] Koenig, H. G., Carey, L. B., & Wortham, J. S. (2023). *Moral Injury: A handbook for military chaplains*. Amazon.co.uk: Books.

[CR32] Litz, B. T., Stein, N., Delaney, E., Lebowitz, L., Nash, W. P., Silva, C., & Maguen, S. (2009). Moral injury and moral repair in war veterans: A preliminary model and intervention strategy. *Clinical Psychology Review,**29*(8), 695–706. 10.1016/j.cpr.2009.07.003 19683376 10.1016/j.cpr.2009.07.003

[CR33] Liuski, T., & Grimell, J. (2022). The professional competence of Finnish and Swedish military chaplains in divergent operational environments. *Uskonto, Katsomus Ja Kasvatus,**2*(1), 74–96. 10.63389/ukk.115216

[CR34] Liuski, T., & Ubani, M. (2020). How is military chaplaincy in Europe portrayed in European scientific journal articles between 2000 and 2019? A multidisciplinary review. *Religions,**11*(10), 540. 10.3390/rel11100540

[CR300] Mattson, S., VanderWeele, T. J., Lu, F., Carey, L. B., Cowden, R. G., Fung, E. N., Koenig, H. G., Peteet, J. & Wortham, J. (2025). Moral, Religious, or Spiritual Problem: An Expanded Z Code Diagnostic Category in the DSM-5-TR. *The Journal of Nervous and Mental Disease,**213*(11), 297–304. 10.1097/NMD.0000000000001856 41195647 10.1097/NMD.0000000000001856

[CR35] Moskos, C. C., Williams, J. A., & Segal, D. R. (Eds.). (2000). *The postmodern military: Armed forces after the cold war*. Oxford University Press.

[CR36] Mudde, L., Schuhmann, C., & Jacobs, G. (2025). Engaging in moral learning: Veterans’ perspectives on how the moral dimensions of moral injury are addressed in one-on-one meetings with Dutch military chaplains. *Frontiers in Sociology,**10*, 1488372. 10.3389/fsoc.2025.1488372 40061056 10.3389/fsoc.2025.1488372 PMC11887478

[CR37] Nakashima Brock, R., & Lettini, G. (2012). *Soul Repair. Recovering from Moral Injury after War*. Beacon Press.

[CR38] Pargament, K. I. (2011). *Spiritually integrated psychotherapy: Understanding and addressing the sacred*. The Guilford Press.

[CR39] Pargament, K. I., & Sweeney, P. J. (2011). Building spiritual fitness in the Army: An innovative approach to a vital aspect of human development. *American Psychologist,**66*(1), 58–64. 10.1037/a0021657 21219049 10.1037/a0021657

[CR40] Peuckmann, N. (2022). *In kritischer Solidarität. Eine Theorie der Militärseelsorge*. Evangelische Verlagsanstalt.

[CR41] Pleizier, T., & Schuhmann, C. (2022). How the military context shapes spiritual care interventions by military chaplains. *Journal of Pastoral Care & Counseling,**76*(1), 4–14. 10.1177/15423050221076462 35098792 10.1177/15423050221076462

[CR42] Schuhmann, C., Pleizier, T., Walton, M., & Körver, J. (2023). How military chaplains strengthen the moral resilience of soldiers and veterans: Results from a case studies project in the Netherlands. *Pastoral Psychology,**72*, 605–624. 10.1007/s11089-023-01097-5

[CR43] Shay, J., & Munroe, J. (1998). Group and milieu therapy for veterans with complex posttraumatic stress disorder. In P. A. Saigh & J. D. Bremner (Eds.), *Posttraumatic stress disorder: a comprehensive text* (pp. 391–413). Allyn & Bacon.

[CR44] Stallinga, B. A. (2013). What spills blood wounds spirit: Chaplains, spiritual care, and operational stress injury. *Reflective Practice: Formation and Supervision in Ministry,**33*, 13–31.

[CR45] Stewart, D. W. (2012). Compassion fatigue: What is the level among army chaplains? *Journal of Workplace Behavioral Health,**27*(1), 1–11.

[CR46] Strachan, H. (2006). Morale and modern war. *Journal of Contemporary History,**41*(2), 211–227. 10.1177/0022009406062054

[CR47] van der Brug, W., Hobolt, S. B., & Popa, S. A. (2026). The kids are Alt right? Age, authoritarian attitudes and far-right support in Europe. *Journal of European Public Policy,**33*(2), 469–494. 10.1080/13501763.2025.2488358

[CR48] Verrips, J. (2006). Dehumanization as a double-edged sword. In G. Baumann & A. Gingrich (Eds.), *Grammars of identity/alterity: A structural approach* (pp. 142–154). Berghahn Books.

[CR49] Visser, A., Vähäkangas, A., Baig, N., & Thomsen, K. F. (2025). Chaplaincy in a pluralistic context. *Journal of Health Care Chaplaincy,**31*(4), 239–245. 10.1080/08854726.2025.2569223 41076643 10.1080/08854726.2025.2569223

[CR50] Wortmann, J. H., Eisen, E., Hundert, C., Jordan, A. H., Smith, M. W., Nash, W. P., & Litz, B. T. (2017). Spiritual features of war-related moral injury: a primer for clinicians. *Spirituality in Clinical Practice,**4*(4), 249–261. 10.1037/scp0000140

[CR51] Zimbardo, P. (2008). *The Lucifer effect: Understanding how good people turn evil*. Random House Trade Paperbacks.

