# Correction: At the edge of war: What rapid reinforcement of European defence may mean for military chaplains

**DOI:** 10.1007/s10943-026-02654-x

**Published:** 2026-04-10

**Authors:** Jan Grimell, Alexandra Dierks, Matthias Inniger, Taavi Laanepere, Tatiana Letovaltseva, Tiia Liuski, Laura Mudde, Niklas Peuckmann, Carmen Schuhmann, Janne Aalto, Tabitta Flyger

**Affiliations:** European Research Unit for Military Chaplaincy at Umeå University, Umeå, Sweden

**Correction to: Journal of Religion and Health** 10.1007/s10943-026-02625-2

In the original article, the corrections below are to be updated.

1. The title is corrected to conform with APA-7 standards with a capital following the full colon and thus revised to: "At the edge of war: What rapid reinforcement of European defence may mean for military chaplains".
2. A missing clause and reference is added to the original sentence within the section **Moral injury in war: preventive perspective** which states, "By contrast, there remains relatively limited research on how moral injury functions, and how the concept should be applied, in the midst of ongoing war. As a result, our current understanding of moral injury “in war” remains underdeveloped". This sentence is now extended with the additional words: "…..however progress is being made given the recent recognition of moral problems and subsequently moral injury as a supplementary condition among active military personnel and veterans, which is now noted within the Diagnostic and Statistical Manual for Mental Health Disorders (DSM-5-TR; Mattson et al., 2025).
3. The additional reference is added to the reference list:
  Mattson, S., VanderWeele, T.J., Lu, F., Carey, L.B., Cowden, R.G., Fung, E.N., Koenig, H.G., Peteet, J. & Wortham, J. (2025). Moral, Religious, or Spiritual Problem: An Expanded Z Code Diagnostic Category in the DSM-5-TR. *The Journal of Nervous and Mental Disease*, *213*(11), pp.297-304. 10.1097/NMD.0000000000001856

